# MiT translocation renal cell carcinomas: two subgroups of tumours with translocations involving 6p21 [t (6; 11)] and Xp11.2 [t (X;1 or X or 17)]

**DOI:** 10.1186/2193-1801-3-245

**Published:** 2014-05-13

**Authors:** Milan Hora, Tomáš Ürge, Ivan Trávníček, Jiří Ferda, Zdeněk Chudáček, Tomáš Vaněček, Michal Michal, Fredrik Petersson, Naoto Kuroda, Ondřej Hes

**Affiliations:** Department of Urology Faculty Hospital, E. Beneše 13, Pilsen, 305 99 Czech Republic; Department of Radiology, Pilsen, Czech Republic; Department of Pathology, Faculty Hospital in Pilsen, Pilsen, Czech Republic; Department of Pathology, National University Health System, Singapore, Singapore; Faculty of Medicine in Pilsen, Charles University in Prague, Prague, Czech Republic; Department of Diagnostic Pathology, Kochi Red Cross Hospital, Kochi, Japan

## Abstract

**Introduction:**

MiT translocation renal cell carcinomas (TRCC) predominantly occur in younger patients with only 25% of patients being over 40 years. TRCC contains two main subgroups with translocations involving 6p21 or Xp11.2. Herein we present 10 cases.

**Materials:**

Eight cases were treated at main author’s institution (identified among 1653 (0.48%) cases of kidney tumours in adults). Two cases were retrieved from the Pilsen (CZ) Tumour Registry.

**Results:**

Six cases were type Xp11.2 and four 6p21; 7 female, 3 male patients; Xp11.2 4:2, 6p21 3:1. The mean age 49 years (range: 21–80), 5 patients (50%) over 40 years. The mean age of the group with Xp11.2 TRCCs was 55 (median 51) and 6p21 41 (32) years. One female with a 6p21 tumour (24 years) underwent nephrectomy at 4 months of pregnancy. Stage (UICC, 7th ed. 2009) was 5xI, 3xIII, 2xIV. The mean size of tumour was 80 (40–165) mm. The mean follow-up was 33.2 (1–92) months. In patients with 6p21 tumours, one (25%) died after 3 months due to widely metastatic disease. In patients with Xp11.2 tumours, 3 (50%) succumbed due to metastatic disease (range 1–8 months). Three patients with Xp11.2 are alive at 7, 52 and 92 months of follow-up, were diagnosed at early stage (T1a).

**Conclusion:**

TRCCs were more common in females. Patient with 6p21 tumours were younger than those with Xp11.2. Both types have definitive malignant potential Type Xp11.2 seems to be a more aggressive neoplasm than 6p21. The case with metastatic 6p21 tumour is the 4th case described in the English literature.

## Introduction

MiT translocation renal cell carcinomas (TRCC) constitute a group of recently described rare kidney tumours. These tumours predominantly occur in younger patients with only about 25% affecting patients over 40 year of age (see Table 1). TRCC contains two main subgroups: Srigley et al. 2013 Tumours with translocation 6p21 [t (6;11)] have characteristic histopathological features and imunohistochemical properties and have been labelled “rosette forming HMB45 positive renal tumour” in addition to “TFEB RCC”. Hora et al. 2008 The second subgroup is composed of tumours with translocations involving Xp11.2 [t (X; 1 or X or 17)]. TRCC Xp11.2 is included in the 2004 WHO renal tumours classification already. In the ISUP (International Society of Urological Pathology) Vancouver classification of renal neoplasia Srigley et al. 2013 these tumours have been added as a new subgroup of RCC: “MiT family TRCC” with two subgroup – Xp11 TRCC and t (6;11) RCC.

**Table 1:** **Review of literature about MiT TRCC since 2007**

Author	Year	No of patients			Age				Sex	Size	Staging (at presentation/maximal by follow-up)				Surgery	Oncological therapy	Survival
		Together	TRCC		Average	Range	Over 40		F:M		I	II	III	IV	PNE/NE		
			Xp11.2	6p21			Total	%		average, in mm	T1N0M0	T2N0M0	T3N0M0, T1-3N1M0	T4NXM0, TXN2M0, TXNXM1			
Argani	2007	28	28	0	37.2	22 - 78	7	25.0%	22 : 6	6.8	9	3	2	14	UK	in 1 case, imuno- and RT	6:3 malignant vs bening course
Rais-Bahrami	2007	1	1		6	NA	0	0.0%	0 : 1	1.5	1				0/1	TKI, progression, death in 8 ms	generalisation 17 ys later
LaGrande	2007	1	1		63	NA	1	100.0%	1 : 0	3	1						2 ys without sign of recurrence
Franzini	2007	1	1		79	NA	1	100.0%	0 : 1	12			1			none	metastases in 1 m
Hora	2008	2	2	0	57	34-80	1	50.0%	1:1	46	1		1			none	one died 1 mfor generalisation
Camparo	2008	3423	29	2	24.6	0.9-64	3	9.7%	18:13	69	12	2	7	10	4/27		FU 29.5 ms, 21 NoR, 5 died, 3 metastases, 2 lost for FU
Hora	2009	15000	0	3	28.3	22-39	0	0.0%	2:01	62	2	1			2/1	none	no recurrence
Suárez-Vilela	2009	1	0	1	22.0	22	0	0.0%	0 : 1	large		1			0/1		no recurrence
Koie	2009	1	1		28.0	NA	0	0.0%	0 : 1	85		1			0/1	excision of local recurrence, cytokins	died in 24 ms
Armah	2009	1	1		26.0	NA	0	0.0%	1 : 0	75		1			1/0	none	pregnant, 27 ms without recurrence
Kuroda	2010	1	1		73.0		1	100.0%	1 : 0	20	1						Unknown
Jing	2010	1	1		12.0	NA	0	0.0%	0 : 1	60		1				Ch, RT	Alive in 17 ms
Choueiri	2010	15	15		41.0	18-65	UK	UK	12:3	UK	2	1	8	8	0/12	VEGF-targeted therapy	
Ishihara	2011	1		1	45.0	NA	1	100.0%	0 : 1	70				1	0/1	ChT, RT, temsirolimus	in 8 ms generalisation
Liu	2011	1	1		27.0	NA	0	0.0%	1 : 0	100				1	0/1	metastasectomy, gemcitabin, sunitinib	partial response to sunitinib
Nelius	2011	1	1		19.0	NA	0	0.0%	0 : 1	115				1	1/0	ChT, sorafenib, temsirolimus, bavacizumab, RT	died in 3 ms
Numakura	2011	1	1		43.0	NA	1	100.0%	1 : 0	100			1		1/0	sunitinib - PR for over 3years	lung metatases 2 ys from surgery
Kato	2011	1	1	0	18.0	18	0	0.0%	0:1	41	1				0/1	none	UK
Klatte	2012	848	2	0	NA	5 and 42	1	50.0%	1 : 1	100	0	1	0	1	0/2	dendritics cells and interferon-alfa-2a (CR)	4.5 and 8 ms
Morii	2012	1	1	0	38.0	38	0	0.0%	1 : 0	75	0	0	0	1	1/0	imunotherapy, sunitinib, sorafenib, mTOR I	died in 16 ms
Rao	2012	7	0	7	30.6	21-37	0	0.0%	4 : 3	51	5	2	0	0	UK	none	NED in any case
Inamura	2012	200	0	3	47.0	37-57	2	66.7%	0:3	UK	UK	UK	UK	UK	UK		one aggresive course
Arnoux	2012	170	4	0	UK	UK	4	100.0%	UK	UK	0	0	1	3	4/0	UK	one died, 3 others progression
Dang	2012	9	9	0	29.6	18-45	1	11.1%	3:6	58	UK	UK	UK	UK	5-Mar	ChT 4 cases, RT 2 cases, 2 excision of retroperitoneal recurrence	2 died, 1 metastatic disease, 2 local recurrence
Komai	2013	443	7	0	42.0	15-59	5	71.4%	3:4	83	3 (Hora et al. 2008)	1 (0)	0 Srigley et al. (2013)	2 Klatte et al. (2012)	0/7	UK	5 of 7 (71.4%) generalisation
Gaillot-Durand	2013	92	2	0	UK	UK	UK	UK	UK	UK	UK	UK	UK	UK	UK	UK	UK
Together		20255	110	17			29	22.8%			35	14	21	40			
			127								32%	13%	19%	36%			

Papers dealing with TRCCs have been published mostly by pathologists and geneticists. It is difficult and time consuming to get clinically relevant data useful for daily urological practice. We present 10 cases of TRCC collected from the whole Czech Republic (10 million of inhabitants) focusing on data important from to point of view of practicing urologists.

## Material and methods

During 2001 to 2012, 1653 kidney tumours were surgically treated at the urological department of the main author. Eight of them were TRCC (0.48%). Two more cases were identified in the tumour registry of the Department of Pathology, Faculty Hospital, Pilsen, CZ. The registry includes over 16000 cases of renal tumours, of which a significant component is international consult cases. Due to easy access to clinical data including CT and to avoid ethical problems with approving the study in different countries, only cases from CZ were included. These ten cases are presented in detail. Five cases have previously been published Hora et al. 2008; Hora et al. 2009. Extended follow-up information is provided for these cases in this paper. These cases are: two TRCC Xp.11 (in Table 2 cases 2 and 5) Hora et al. 2008 and three TFEB TRCC (in Table 2 cases 1, 3, 4) Hora et al. 2009. The morphological diagnosis was supported by immunohistochemical examination. In 1 of 4 cases of Xp11.2 TRCC, morphological and immunohistochemical results were extended by FISH analysis of TFE3 break. See Table 3. In one case, the diagnosis was verified. The other 4 cases were not analysable due to low quality of DNA. Among 4 cases of 6p21, three were morphological and immunohistochemical analysis supported by FISH and RT PCR (reverse transcription polymerase chain reaction) analysis. The presence of the translocation t (6;11) (Alpha-TFEB) was confirmed in these 3 analysed cases. In one case (percutaneous biopsy only), the diagnosis was established without molecular genetic confirmation, i.e. based exclusively on morphology and results of immunohistochemistry.

**Table 2:** **Results**

No	Age			Sex	Date of surgery	Side	Type of surgery	Note	AE	LAE	Size of tumour (mm)	Staging					Grading by Fuhrmann	Follow-up in months
	Both TRCCs	TRCC Xp11.2	TRCC 6p21									pT	cN	cM	Stage at diagnosis	Stage at the last follow-up		
10	34.5	34.5		F	1/2/2013	L	Biopsy under CT	T3aN2M1 - metastasis to liver, pubic bone, cisplatina and temsirolimus no effect	0	0	96	3a	2	1	4	4	UK	8
9	77.6		77.6	F	10/3/2012	L	ONE, AE, LND	Cytoreductive NE of bulky tumour (1850 g), lung metastases	1	1	165	3a	c1p0	p1*	3	4	3	3
8	75.3	75.3		F	21.11 (November). 2012	R	OR		0	0	33	1a	0	0	1	0	3	7
7	42.3	42.3		M	1/1/2011	L	ONE	Multiple skeletal metastases, liver, lung	0	0	40	3a	1	1	4	4	3	8
6	60.2	60.2		F	3/2/2011	L	LNE		0	0	83	1b	0	0	1	0	3	27
5	34.9	34.9		F	1/10/2008	L	OR	Followed like a cystic renal leasion, control MRI Bosniak IV, published formerly Hora et al. (2008)	0	0	32	1a	0	0	1	0	3	64
4	24.4		24.4	F	2/23/2007	R	ONE	Published formerly Hora et al. (2009)	0	0	128	3a	0	0	3	0	1	76
3	39.3		39.3	F	6/11/2007	R	OR	Published formerly Hora et al. (2009)	0	0	10	1a	0	0	1	0	1	47
2	80.3	80.3		M	28 Dec 2007	L	ONE	Published formerly Hora et al. (2008)	0	0	130	3a	0	0	3	3	3	1
1	21.4		21.4	M	5/1/2005	R	ONE	Published formerly Hora et al. (2009)	0	0	40	1a	0	0	1	0	1	92
	49.0	54.6	40.7	average						75.7							33.2	
	22.4	20.3	25.8	STDEV.S						52.4							34.1	
	21.4	34.5	21.4	MIN						10.0							0.6	
	80.3	80.3	77.6	MAX						165.0							92.4	
	40.8	51.2	31.9	MEDIAN														

Notes, abbreviations: Black windows – the patient died due to tumour.

AE – adrenalectomy, ONE – open nephrectomy, LNE - laparoscopic NE, OR – open resection, LND lymph node dissection, UK unknown, STDE.S – standard deviation, MIN – minimal value, MAX maximal value, NA – DNA from specimen not analysable due to low quality, ND – not done, pos. – positive, TRCC Translocation renal cell carcinoma.

**Figure 1:**
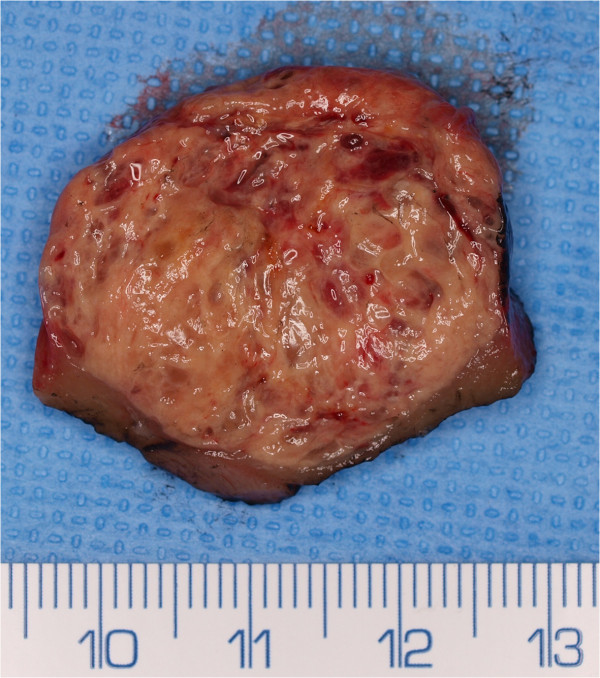
**Dissected specimen at operation: woman, 75-year-old, a tumour of the right kidney.** In table case No. 5. Tumour was on CT spheroid, 34 mm in maximal diameter, relatively homogenous, native density13-26HU, postcontrast density (venous phase) 15–43 HU. R was performed. A dissected specimen at operation: ochre-orange relatively homogenous spheroid tumour very different from clear renal cell carcinoma, maybe a little similar to any papillary RCC. Histological diagnosis: Translocation carcinoma Xp11.2, subtype ASPL-TFE3, verified genetically.

**Figure 2:**
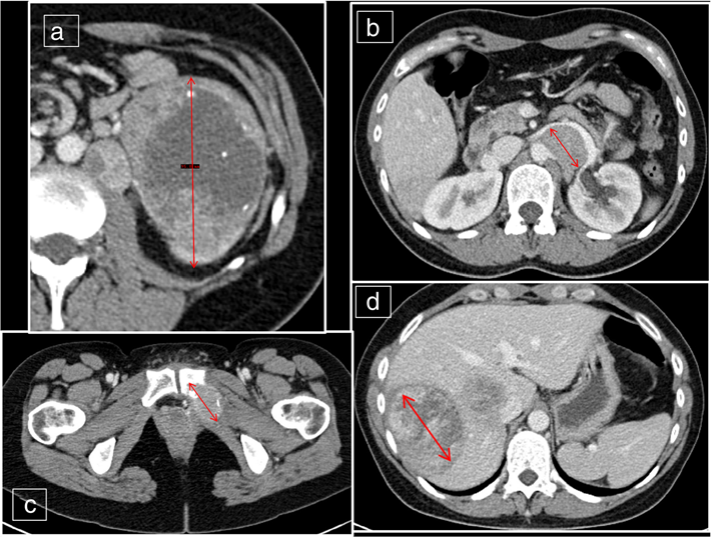
**Woman 34-year-old, tumour of the left kidney (a) T3aN2M1, metastases to the paraaortal lymph nodes (b), left pubic bone (c), liver (d).** Biopsy Translocation carcinoma Xp11.2. In Table 2 case No. 10.

**Figure 3:**
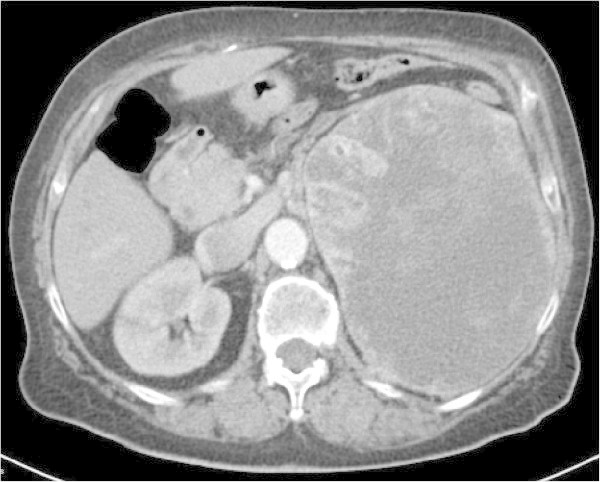
**Postcontrast CT: woman 78-year-old, left kidney tumour T3aN0M1 (metastases to lung), maximal diameter of tumour 172 mm.** In Table 2, case No. 9. She underwent cytoreductive nephrectomy, specimen 1850 g. Histology TRCC 6p21. She died in 3 months. The forth case of aggressive TRCC 6p21 described in literature.

Cases 1, 9 and 10 see Figures 1, 2 and 3.

**Table 3:** **Types of translocation renal cell carcinoma Xp11.2 Hora et al. (** 2008 **)**

Translocation	Fusion of genes
t (X;1) (p11.2; q21)	*PRCC* and *TFE3*
t (X;1) (p11.2; p34)	*PSF* and *TFE3*
t (X;17) (p11.2; q25)	*ASPL* (known as *RCC17* or *ASPSCR1* as well) and *TFE3*
t (X;17) (p11.2; q23)	*CLTC* (*Clathrine*) and *TFE3*
inv (X) (p11.2; q12)	*NonO* (p54^nrb^) a *TFE3*
T (X;3) (p11.2; q23)	Unknown

## Results

The results are summarised in the Table 2. Selected cases, see Figures 1, 2 and 3. Based on these, we can conclude that TRCCs were more common in females (70%). Patient with TRCC type 6p21 were younger than those with Xp11.2 TRCC (average 40.7 ± 25.8 vs. 54.6 ± 20.3 years, median 31.9 vs. 51.2). The biological behaviour of the two main group of MiT TRCC is probably different. Type Xp11.2 TRCC is a more aggressive neoplasm with (malignant course in 3/6 cases – 50%). In four patients with 6p21 TRCC, one (25%) died due to generalisation.

## Discussion

MiT (F) (microphtalmia transcription factor) TRCCs most likely originate from the proximal tubule of the nephron. TRCCs are found predominantly in children and young adults, but are increasingly being recognized in adults. These tumours are characterized by the occurrence of recurrent chromosomal translocations, which result in disruption and fusion of either the *TFE3* or *TFEB* genes, both members of the MiT family of basic helix-loop-helix/leucine-zipper transcription factor genes. Hence the name MiT translocation subgroup of RCCs. The MiT family (MiTF/TFE family) includes TFE3, TFEB, and also TFEC and MiTF transcription factors Srigley et al. 2013.

In clinical practise, we most commonly encounter TRCC TFE3 (divided in at least 6 subtypes, see Table 3) and TFEB type. We have identified 110 published cases of Xp11.2 TRCC in the English literature since 2007 to October 2013. See Table 1. The *TFE3* gene is located on the X chromosome, locus p11.2. Translocations result in fusions of the *TFE3* gene with several other genes which are listed in Table 3. Question The correct diagnosis is reached through a combination of histomorphology, immunohistochemistry and, in selected cases, molecular genetic studies. Since the translocations lead to overexpression of the TFE3 protein, immunohistochemical staining for TFE3 is widely used as a surrogate marker for the Xp11.2 translocation Klatte et al. 2012 Komai et al. Komai et al. 2009 described 7 cases of TRCC Xp11.2, 4 of them were diagnosed cytogenetically, 3 by immunohistochemistry only. Argani et al. 2007 confirmed genetically in group of 28 Xp11 TRCC only three. But Klatte et al. 2012 found in 17 TFE3 positive tumours Xp11.2 translocation in only 2 cases and they recommended making diagnosis of Xp.11.2 translocation RCC only genetically. Other group verified Xp11.2 by RT-PCR 2 cases in 6 TFE3 positive Gaillot-Durand et al. 2013 TFE3 negative tumours on immunohistochemistry can be FISH positive Rao et al. 2013. Our ten cases of TRCC were diagnosed by morphology and immunohistochemistry and only one verified genetically because the limitation of the quality of DNA. But we hope that modern reliable imunohistochemical staining can be very precise and more exact. Sensitivity and specificity of TFE3 tests are now being discussed by pathologists. Manual overnight labelling seems to be more precise than automated immunostainer with 30 min incubation Gaillot-Durand et al. 2013. However precise histological diagnosis verified by cytogenetic studies is missing in some cases and it can be source of bias of this study.

There is little information on clinically relevant data when dealing with patients with Xp11.2 TRCC. The tumours most commonly affect patient under 45 year up to 15% Kuroda et al. 2012. In some previous studies data on the radiological features (CT and MRI) of Xp11 TRCC have been presented. These have failed to identify any specific radiological features of this tumour Liu et al. 2013;Koo et al. 2013; Kato et al. 2011).

Xp11.2 RCC is a biologically aggressive neoplasm with a bad prognosis and previously published data suggest that the prognosis may be even worse in men over 50 years Kuroda et al. 2012; Arnoux et al. 2012. In our group of 6 cases, the ages were similar in patients with aggressive vs. with benign clinical course 52.4 vs. 56.8 years). The oncological therapy is reviewed in Table 1. The same oncological treatment protocols as for clear cell RCC have been used. No firm conclusions regarding the efficacy can be drawn based on this data. In our group, 2/3 of cases have a fatal course in relatively short follow-up.

The other (rare) but more commonly encountered MiT TRCC is “CC with t (6;11) translocation” (abbreviated “6p21 TRCC” or “t (6;11) TRCC”). Owing to the characteristic histopathological features and imunohistochemical properties of this tumour, it has been termed “osette-like forming, HMB45-positive renal tumour” Hora et al. 2009. An alternative designation/term that is used is “TFEB RCC”. This tumour harbours translocations involving the transcription factor EB (TFEB) and *Alpha* (the latter also known as *MALATI*). Genetically, TFEB RCC has been characterized by the fusion of the 5’ portion of *Alpha*, also known as *MALATI* (Genbank accession number AF203815), an intronless gene mapped at 11q12, with *TFEB* at 6p21 Inamura et al. 2012; Rao et al. 2012 with fewer than 30 cases reported to date Hora et al. 2009; Inamura et al. 2012; Rao et al. 2012; Camparo et al. 2008; Suarez-Vilela et al. 2011. First 11 cases were reviewed by Hora et al. 2009, cases published since 2007 are summarised in Table 1. The morphology of TFEB TRCC is distinctive and the diagnosis can be established based on a combination of histopathological examination in conjunction with immunohistochemistry. Role of molecular genetic studies is not as crucial as in Xp11.2 TRCC Hora et al. 2009. The malignant potential is low. Only three cases with aggressive behaviour have been published (approximately 10%) Inamura et al. 2012; Pecciarini et al. 2007; Ishihara et al. 2011. We add one more case with aggressive clinical course.

## Conclusion

TRCCs were more common in females. Patient with 6p21 were younger than those with Xp11.2. Both types have definitive malignant potential, type Xp11.2 TRCC seem to be more aggressive neoplasm. Metastatic 6p21 is 4th case described in literature. From a clinical point of view, subclassification of TRCCs is of utmost clinical relevance.

Citations of articles used in the table: Argani et al. 2007 Rais-Bahrami et al. 2007 LaGrange et al. 2007 Franzini et al. 2007 Hora et al. 2008 Camparo et al. 2008 Hora et al. 2009 Suarez-Vilela et al. 2011, Koie et al. 2009 Armah et al. 2009 Kuroda et al. 2010 Choueiri et al. 2010 Ishihara et al. 2011 Liu et al. 2011 Nelius et al. 2011 Numakura et al. 2011, Kato et al. 2011 Klatte et al. 2012 Morii et al. 2012 Rao et al. 2012 Inamura et al. 2012 Arnoux et al. 2012 Komai et al. 2009 Gaillot-Durand et al. 2013.

## Abbreviations

ChT: Chemotherapy
m (s): month(−s)
ImTOR: Inhibitors (temsirolimus everolimus)
NED: No evidence of disease
NoR: Nor recurrence or metastasis
PR: Partial response
RT: Radiotherapy
TKI: Tyrosine kinase inhibitors
Y (s): Year (−s).

## References

[CR1] Argani P, Olgac S, Tickoo SK, Goldfischer M, Moch H, Chan DY, Eble JN, Bonsib SM, Jimeno M, Lloreta J, Billis A, Hicks J, De Marzo AM, Reuter VE, Ladanyi M (2007). Xp11 translocation renal cell carcinoma in adults: expanded clinical, pathologic, and genetic spectrum. Am J Surg Pathol.

[CR2] Armah HB, Parwani AV, Surti U, Bastacky SI (2009). Xp11.2 translocation renal cell carcinoma occurring during pregnancy with a novel translocation involving chromosome 19: a case report with review of the literature. Diagn Pathol.

[CR3] Arnoux V, Long JA, Fiard G, Pasquier D, Bensaadi L, Terrier N, Rambeaud JJ, Descotes JL (2012). Xp11.2 translocation renal carcinoma in adults over 50years of age: About four cases. Prog Urol.

[CR4] Camparo P, Vasiliu V, Molinie V, Couturier J, Dykema KJ, Petillo D, Furge KA, Comperat EM, Lae M, Bouvier R, Boccon-Gibod L, Denoux Y, Ferlicot S, Forest E, Fromont G, Hintzy MC, Laghouati M, Sibony M, Tucker ML, Weber N, Teh BT, Vieillefond A (2008). Renal translocation carcinomas: clinicopathologic, immunohistochemical, and gene expression profiling analysis of 31 cases with a review of the literature. Am J Surg Pathol.

[CR5] Choueiri TK, Lim ZD, Hirsch MS, Tamboli P, Jonasch E, McDermott DF, Dal Cin P, Corn P, Vaishampayan U, Heng DY, Tannir NM (2010). Vascular endothelial growth factor-targeted therapy for the treatment of adult metastatic Xp11.2 translocation renal cell carcinoma. Cancer.

[CR6] Franzini A, Picozzi SC, Politi PL, Barana L, Bianchi F, Alfano G, Gatti G, Fanciullacci F, Gariboldi M, Strada M, Leone BEA (2007). case of renal cancer with TFE3 gene fusion in an elderly man. Clinical, radiological and surgical findings. Urol Int.

[CR7] Gaillot-Durand L, Chevallier M, Colombel M, Couturier J, Pierron G, Scoazec JY, Mege-Lechevallier F (2013). Diagnosis of Xp11 translocation renal cell carcinomas in adult patients under 50years: interest and pitfalls of automated immunohistochemical detection of TFE3 protein. Pathol Res Pract.

[CR8] Hora M, Hes O, Eret V, Urge T, Klečka J, Ferda J, Chudáček Z, Vanček T, Michal M (2008). Translokační renální karcinom Xp11. 2 typu ASPL/TFE3. Ces Urol.

[CR9] Hora M, Hes O, Urge T, Eret V, Klecka J, Michal M (2009). A distinctive translocation carcinoma of the kidney [“rosette-like forming” t (6;11), HMB45-positive renal tumor]. Int Urol Nephrol.

[CR10] Inamura K, Fujiwara M, Togashi Y, Nomura K, Mukai H, Fujii Y, Yamamoto S, Yonese J, Fukui I, Ishikawa Y (2012). Diverse fusion patterns and heterogeneous clinicopathologic features of renal cell carcinoma with t (6;11) translocation. Am J Surg Pathol.

[CR11] Ishihara A, Yamashita Y, Takamori H, Kuroda N (2011). Renal carcinoma with (6;11) (p21; q12) translocation: report of an adult case. Pathol Int.

[CR12] Kato H, Kanematsu M, Yokoi S, Miwa K, Horie K, Deguchi T, Hirose Y (2011). Renal cell carcinoma associated with Xp11.2 translocation/TFE3 gene fusion: radiological findings mimicking papillary subtype. J Magn Reson Imaging.

[CR13] Klatte T, Streubel B, Wrba F, Remzi M, Krammer B, de Martino M, Waldert M, Marberger M, Susani M, Haitel A (2012). Renal cell carcinoma associated with transcription factor E3 expression and Xp11.2 translocation incidence, characteristics, and prognosis. Am J Clin Pathol.

[CR14] Koie T, Yoneyama T, Hashimoto Y, Kamimura N, Kusumi T, Kijima H, Ohyama C (2009). An aggressive course of Xp11 translocation renal cell carcinoma in a 28-year-old man. Int J Urol.

[CR15] Komai Y, Fujiwara M, Fujii Y, Mukai H, Yonese J, Kawakami S, Yamamoto S, Migita T, Ishikawa Y, Kurata M, Nakamura T, Fukui I (2009). Adult Xp11 translocation renal cell carcinoma diagnosed by cytogenetics and immunohistochemistry. Clin Cancer Res.

[CR16] Koo HJ, Choi HJ, Kim MH, Cho KS (2013). Radiologic-pathologic correlation of renal cell carcinoma associated with Xp11.2 translocation. Acta Radiol.

[CR17] Kuroda N, Katto K, Tanaka Y, Yamaguchi T, Inoue K, Ohara M, Mizuno K, Hes O, Michal M, Lee GH (2010). Diagnostic pitfall on the histological spectrum of adult-onset renal carcinoma associated with Xp11.2 translocations/TFE3 gene fusions. Med Mol Morphol.

[CR18] Kuroda N, Mikami S, Pan CC, Cohen RJ, Hes O, Michal M, Nagashima Y, Tanaka Y, Inoue K, Shuin T, Lee GH (2012). Review of renal carcinoma associated with Xp11.2 translocations/TFE3 gene fusions with focus on pathobiological aspect. Histol Histopathol.

[CR19] LaGrange CA, Lele SM, Strup SE (2007). Renal cell carcinoma associated with TFE3 gene fusion in an elderly woman. Urology.

[CR20] Liu YC, Chang PM, Liu CY, Yang CY, Chen MH, Pan CC, Chen MH (2011). Sunitinib-induced nephrotic syndrome in association with drug response in a patient with Xp11.2 translocation renal cell carcinoma. Jpn J Clin Oncol.

[CR21] Liu K, Xie P, Peng W, Zhou Z (2013). Renal carcinomas associated with Xp11.2 translocations/TFE3 gene fusions: Findings on MRI and computed tomography imaging. J Magn Reson Imaging.

[CR22] Morii A, Fujiuchi Y, Nomoto K, Komiya A, Fuse H (2012). Rapidly progressing renal cell carcinoma associated with Xp11.2 translocations: a case report. J Med Case Rep.

[CR23] Nelius T, Al-Khalil I, Vordermark J, Rinard-Holden K, Cammack T, Mamlok V, Filleur S (2011). TFE3 Translocation-Associated Renal Cell Carcinoma Presenting as Avascular Necrosis of the Femur in a 19-Year-Old Patient: Case Report and Review of the Literature. Case Rep Med.

[CR24] Numakura K, Tsuchiya N, Yuasa T, Saito M, Obara T, Tsuruta H, Narita S, Horikawa Y, Satoh S, Habuchi TA (2011). case study of metastatic Xp11.2 translocation renal cell carcinoma effectively treated with sunitinib. Int J Clin Oncol.

[CR25] Pecciarini L, Cangi MG, Lo Cunsolo C, Macri E, Dal Cin E, Martignoni G, Doglioni C (2007). Characterization of t (6;11) (p21; q12) in a renal-cell carcinoma of an adult patient. Genes Chromosomes Cancer.

[CR26] Rais-Bahrami S, Drabick JJ, De Marzo AM, Hicks J, Ho C, Caroe AE, Argani P (2007). Xp11 translocation renal cell carcinoma: delayed but massive and lethal metastases of a chemotherapy-associated secondary malignancy. Urology.

[CR27] Rao Q, Liu B, Cheng L, Zhu Y, Shi QL, Wu B, Jiang SJ, Wang Y, Wang X, Yu B, Zhang RS, Ma HH, Lu ZF, Tu P, Wang JD, Zhou XJ (2012). Renal cell carcinomas with t (6;11) (p21; q12): A clinicopathologic study emphasizing unusual morphology, novel alpha-TFEB gene fusion point, immunobiomarkers, and ultrastructural features, as well as detection of the gene fusion by fluorescence in situ hybridization. Am J Surg Pathol.

[CR28] Rao Q, Williamson SR, Zhang S, Eble JN, Grignon DJ, Wang M, Zhou XJ, Huang W, Tan PH, Maclennan GT, Cheng L (2013). TFE3 break-apart FISH has a higher sensitivity for Xp11.2 translocation-associated renal cell carcinoma compared with TFE3 or cathepsin K immunohistochemical staining alone: expanding the morphologic spectrum. Am J Surg Pathol.

[CR29] Srigley JR, Delahunt B, Eble JN, Egevad L, Epstein JI, Grignon D, Hes O, Moch H, Montironi R, Tickoo SK, Zhou M, Argani P (2013). ISUP Renal Tumor Panel. The International Society of Urological Pathology (ISUP) Vancouver Classification of Renal Neoplasia. Am J Surg Pathol.

[CR30] Suarez-Vilela D, Izquierdo-Garcia F, Mendez-Alvarez JR, Miguelez-Garcia E, Dominguez-Iglesias F (2011). Renal translocation carcinoma with expression of TFEB: presentation of a case with distinctive histological and immunohistochemical features. Int J Surg Pathol.

